# 4,4′-(Propane-1,3-di­yl)dipyridinium tetra­chloridonickelate(II)

**DOI:** 10.1107/S1600536809048843

**Published:** 2009-11-21

**Authors:** Zhong-Xiang Du, Ju-Ping Qu

**Affiliations:** a Department of Chemistry, Luoyang Normal University, Luoyang, Henan 471022, People’s Republic of China; b Department of Chemical Engineering and Environmental Engineering, Jiaozuo University, Jiaozuo, Henan 450042, People’s Republic of China

## Abstract

The title compound, (C_13_H_16_N_2_)[NiCl_4_] or (H_2_bpp)·NiCl_4_ [bpp is 1,3-bis­(4-pyrid­yl)propane], is isostructural with its already reported Cu, Zn and Hg analogues. The structure consists of a doubly charged (H_2_bpp)^2+^ cation and a tetra­hedral [NiCl_4_]^2−^ dianion. Both pyridyl N atoms are protonated and form a (H_2_bpp)^2+^ cation which adopts an *anti*–*anti* conformation with a dihedral angle of 6.287 (7)° between the pyridyl rings. The two pyridyl N atoms are both involved in strong N—H⋯Cl hydrogen bonds, which link both units into a dimer.

## Related literature

For the isostructural Cu, Zn and Hg analogues, see: Kao & Chen (2004 ▶).
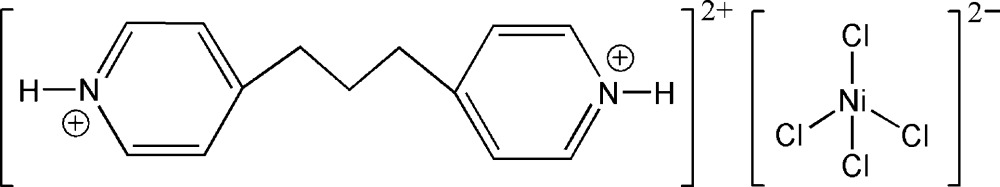


## Experimental

### 

#### Crystal data

(C_13_H_16_N_2_)[NiCl_4_]
*M* *_r_* = 400.79
Monoclinic, 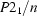

*a* = 7.2358 (7) Å
*b* = 20.773 (2) Å
*c* = 11.1246 (11) Å
β = 90.627 (1)°
*V* = 1672.1 (3) Å^3^
*Z* = 4
Mo *K*α radiation
μ = 1.79 mm^−1^
*T* = 294 K
0.36 × 0.25 × 0.24 mm

#### Data collection

Bruker APEXII CCD area-detector diffractometer
Absorption correction: multi-scan (*SADABS*; Sheldrick, 1996 ▶) *T*_min_ = 0.565, *T*_max_ = 0.673
12584 measured reflections
3107 independent reflections
2556 reflections with *I* > 2σ(*I*)
*R*_int_ = 0.026

#### Refinement

*R*[*F* ^2^ > 2σ(*F* ^2^)] = 0.029
*wR*(*F* ^2^) = 0.045
*S* = 1.90
3107 reflections
181 parameters
H-atom parameters constrained
Δρ_max_ = 0.36 e Å^−3^
Δρ_min_ = −0.45 e Å^−3^

### 

Data collection: *APEX2* (Bruker, 2004 ▶); cell refinement: *SAINT* (Bruker, 2004 ▶); data reduction: *SAINT*; program(s) used to solve structure: *SHELXS97* (Sheldrick, 2008 ▶); program(s) used to refine structure: *SHELXL97* (Sheldrick, 2008 ▶); molecular graphics: *SHELXTL* (Sheldrick, 2008 ▶); software used to prepare material for publication: *SHELXTL*.

## Supplementary Material

Crystal structure: contains datablocks global, I. DOI: 10.1107/S1600536809048843/bg2307sup1.cif

Structure factors: contains datablocks I. DOI: 10.1107/S1600536809048843/bg2307Isup2.hkl

Additional supplementary materials:  crystallographic information; 3D view; checkCIF report

## Figures and Tables

**Table 1:** Hydrogen-bond geometry (Å, °)

*D*—H⋯*A*	*D*—H	H⋯*A*	*D*⋯*A*	*D*—H⋯*A*
N1—H1*A*⋯Cl1^i^	0.86	2.43	3.150 (2)	142
N2—H2*A*⋯Cl3^ii^	0.86	2.25	3.114 (2)	178

Symmetry codes: (i) 
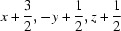
; (ii) 
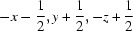
.

**Fig. 1.**
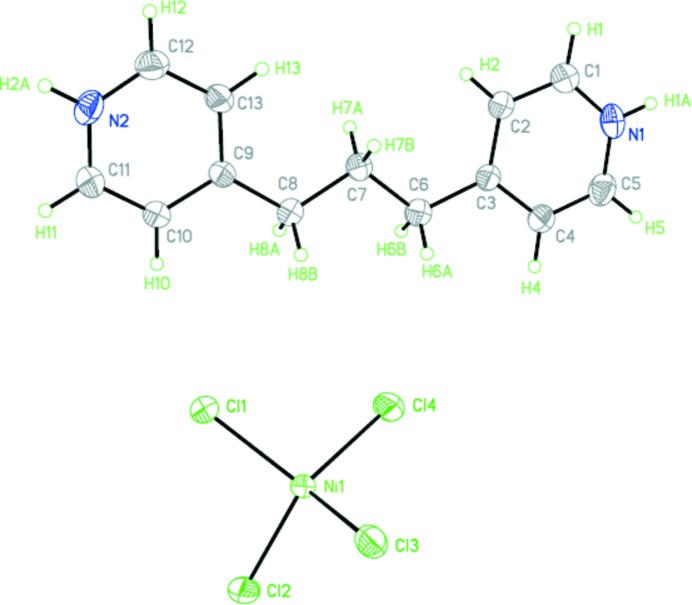
Molecular structure of (I), with displacement ellipsoids drawn at the 30% probability level.

**Fig. 2.**
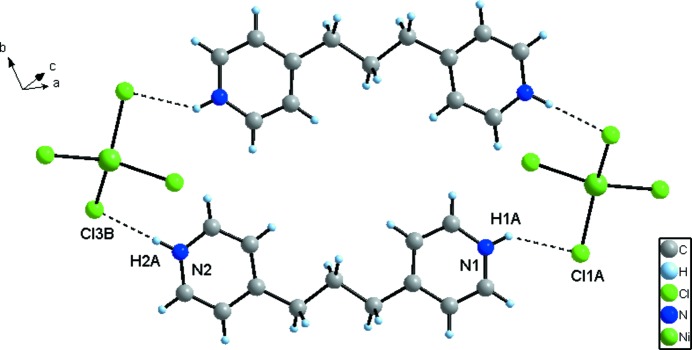
The dimer of (I) formed by N—H···Cl hydrogen bonds showing as dashed lines.

## supplementary crystallographic information

### Comment

The title complex (I) is isostructural with its analogues (H_2_bpp).CuCl_4_ and (H_2_bpp).MCl_4_.H_2_O(*M* = Zn, Hg)(Kao and Chen, 2004), whose structures have been described in detail. The structure consists of a doubly charged (H_2_bpp)^2+^ cation and a tetrahedral NiCl_4_^2-^ dianion (Figure 1). Both pyridyl N atoms are protonated and form a (H_2_bpp)^2+^ cation, which adopts an anti-anti conformations with a dihedral angle of 6.287° between the two pyridyl rings. The two pyridyl N atoms are both involved in strong N—H···Cl hydrogen bonds (Table 1) and link both units into a dimer (Figure 2).

### Experimental

NiCl_2_.6H_2_O (1.0 mmol, 0.237 g), bpp (1.0 mmol, 0.198 g) and oxydiacetatic acid (1.0 mmol, 0.134 g) were dissolved in 20 ml of methanol-H_2_O (*v*/*v*, 1:3). Then the mixture was sealed in a 25 mL Teflon reactor and kept under autogeneous pressure at 403 K for 5 days. After cooling to room temperature at a rate of 6°C.h^-1^, blue block shaped crystals suitable for X-ray diffraction were grown from the filtrate by slow evaporation. Yield: 200 mg (50% based on Ni). Anal. Calcd for C_13_H_16_Cl_4_N_2_Ni(%): C, 38.92; H, 3.99; N, 6.98. Found: C, 38.85; H, 4.03; N, 6.85. CCDC 752249.

### Refinement

H atoms bonded to C and N atoms were positioned geometrically with C—H distance 0.93–0.97Å and N—H distances of 0.8600 Å, and treated as riding atoms, with *U*_iso_(H)=1.2*U*_eq_(C,*N*).

### Crystal data

**Table:**

(C_13_H_16_N_2_)[NiCl_4_]	*F*(000) = 816
*M**_r_* = 400.79	*D*_x_ = 1.592 Mg m^−^^3^
Monoclinic, *P*2_1_/*n*	Mo *K*α radiation, λ = 0.71073 Å
Hall symbol: -P 2yn	Cell parameters from 3858 reflections
*a* = 7.2358 (7) Å	θ = 2.7–25.6°
*b* = 20.773 (2) Å	µ = 1.79 mm^−^^1^
*c* = 11.1246 (11) Å	*T* = 294 K
β = 90.627 (1)°	Block, blue
*V* = 1672.1 (3) Å^3^	0.36 × 0.25 × 0.24 mm
*Z* = 4	

### Data collection

**Table:**

Bruker APEXII CCD area-detector diffractometer	3107 independent reflections
Radiation source: fine-focus sealed tube	2556 reflections with *I* > 2σ(*I*)
graphite	*R*_int_ = 0.026
φ and ω scans	θ_max_ = 25.5°, θ_min_ = 2.7°
Absorption correction: multi-scan (*SADABS*; Sheldrick, 1996)	*h* = −8→8
*T*_min_ = 0.565, *T*_max_ = 0.673	*k* = −24→25
12584 measured reflections	*l* = −13→13

### Refinement

**Table:**

Refinement on *F*^2^	Primary atom site location: structure-invariant direct methods
Least-squares matrix: full	Secondary atom site location: difference Fourier map
*R*[*F*^2^ > 2σ(*F*^2^)] = 0.029	Hydrogen site location: inferred from neighbouring sites
*wR*(*F*^2^) = 0.045	H-atom parameters constrained
*S* = 1.90	*w* = 1/[σ^2^(*F*_o_^2^) + (0.*P*)^2^] where *P* = (*F*_o_^2^ + 2*F*_c_^2^)/3
3107 reflections	(Δ/σ)_max_ < 0.001
181 parameters	Δρ_max_ = 0.36 e Å^−^^3^
0 restraints	Δρ_min_ = −0.45 e Å^−^^3^

### Special details

**Table:**

Geometry. All e.s.d.'s (except the e.s.d. in the dihedral angle between two l.s. planes)are estimated using the full covariance matrix. The cell e.s.d.'s are takeninto account individually in the estimation of e.s.d.'s in distances, anglesand torsion angles; correlations between e.s.d.'s in cell parameters are onlyused when they are defined by crystal symmetry. An approximate (isotropic)treatment of cell e.s.d.'s is used for estimating e.s.d.'s involving l.s. planes.
Refinement. Refinement of *F*^2^ against ALL reflections. The weighted *R*-factor *wR* and goodness of fit *S* are based on *F*^2^, conventional *R*-factors *R* are based on *F*, with *F* set to zero for negative *F*^2^. The threshold expression of *F*^2^ > σ(*F*^2^) is used only for calculating *R*-factors(gt) *etc*. and is not relevant to the choice of reflections for refinement. *R*-factors based on *F*^2^ are statistically about twice as large as those based on *F*, and *R*- factors based on ALL data will be even larger.

### Fractional atomic coordinates and isotropic or equivalent isotropic displacement parameters (Å^2^)

**Table:**

	*x*	*y*	*z*	*U*_iso_*/*U*_eq_	
C1	0.9569 (3)	0.39288 (13)	0.4993 (2)	0.0604 (7)	
H1	0.9940	0.4331	0.5272	0.073*	
C2	0.7956 (3)	0.38678 (11)	0.4357 (2)	0.0534 (7)	
H2	0.7226	0.4228	0.4204	0.064*	
C3	0.7409 (3)	0.32701 (11)	0.3941 (2)	0.0425 (6)	
C4	0.8519 (3)	0.27500 (12)	0.4223 (2)	0.0546 (7)	
H4	0.8166	0.2340	0.3976	0.066*	
C5	1.0119 (4)	0.28295 (13)	0.4855 (2)	0.0611 (7)	
H5	1.0862	0.2476	0.5034	0.073*	
C6	0.5730 (3)	0.31581 (10)	0.3173 (2)	0.0544 (7)	
H6A	0.5005	0.2821	0.3546	0.065*	
H6B	0.6135	0.2995	0.2403	0.065*	
C7	0.4473 (3)	0.37229 (10)	0.2940 (2)	0.0462 (6)	
H7A	0.5157	0.4064	0.2548	0.055*	
H7B	0.4019	0.3888	0.3697	0.055*	
C8	0.2851 (3)	0.35192 (10)	0.2146 (2)	0.0472 (6)	
H8A	0.3340	0.3356	0.1397	0.057*	
H8B	0.2227	0.3165	0.2539	0.057*	
C9	0.1435 (3)	0.40250 (11)	0.1848 (2)	0.0403 (6)	
C10	−0.0087 (3)	0.38588 (11)	0.1149 (2)	0.0497 (7)	
H10	−0.0194	0.3442	0.0853	0.060*	
C11	−0.1429 (3)	0.42979 (12)	0.0891 (2)	0.0551 (7)	
H11	−0.2454	0.4180	0.0429	0.066*	
C12	0.0190 (3)	0.50868 (12)	0.1949 (2)	0.0594 (7)	
H12	0.0274	0.5511	0.2213	0.071*	
C13	0.1564 (3)	0.46566 (11)	0.2227 (2)	0.0519 (7)	
H13	0.2589	0.4791	0.2673	0.062*	
Cl1	−0.06721 (8)	0.21383 (3)	0.12980 (6)	0.05653 (19)	
Cl2	−0.12187 (9)	0.05327 (3)	0.13195 (6)	0.05657 (19)	
Cl3	−0.06868 (9)	0.09147 (3)	0.42665 (6)	0.0606 (2)	
Cl4	0.29570 (8)	0.16495 (3)	0.30041 (6)	0.0633 (2)	
N1	1.0614 (3)	0.34138 (11)	0.52139 (17)	0.0574 (6)	
H1A	1.1641	0.3462	0.5601	0.069*	
N2	−0.1267 (3)	0.48968 (10)	0.13015 (19)	0.0558 (6)	
H2A	−0.2130	0.5170	0.1143	0.067*	
Ni1	0.01874 (4)	0.129429 (13)	0.24398 (3)	0.03832 (9)	

### Atomic displacement parameters (Å^2^)

**Table:**

	*U*^11^	*U*^22^	*U*^33^	*U*^12^	*U*^13^	*U*^23^
C1	0.0602 (19)	0.0548 (17)	0.066 (2)	−0.0016 (15)	−0.0134 (15)	0.0003 (15)
C2	0.0527 (17)	0.0443 (15)	0.0628 (18)	0.0060 (12)	−0.0155 (14)	0.0009 (13)
C3	0.0421 (15)	0.0413 (14)	0.0441 (15)	0.0019 (11)	−0.0031 (12)	0.0036 (12)
C4	0.0569 (17)	0.0454 (15)	0.0613 (18)	0.0086 (13)	−0.0117 (14)	−0.0033 (14)
C5	0.0621 (19)	0.0602 (19)	0.0610 (19)	0.0188 (15)	−0.0051 (15)	0.0026 (16)
C6	0.0508 (17)	0.0451 (15)	0.0669 (19)	0.0019 (12)	−0.0131 (14)	0.0016 (14)
C7	0.0389 (14)	0.0463 (14)	0.0533 (16)	−0.0002 (12)	−0.0049 (12)	0.0005 (13)
C8	0.0482 (16)	0.0417 (14)	0.0515 (16)	0.0019 (12)	−0.0069 (12)	0.0035 (12)
C9	0.0373 (14)	0.0404 (14)	0.0433 (15)	−0.0034 (11)	−0.0009 (11)	0.0003 (12)
C10	0.0482 (16)	0.0410 (15)	0.0597 (18)	−0.0033 (12)	−0.0107 (13)	−0.0013 (13)
C11	0.0455 (16)	0.0567 (17)	0.0627 (19)	−0.0034 (14)	−0.0103 (13)	0.0016 (15)
C12	0.0577 (18)	0.0470 (16)	0.073 (2)	0.0026 (14)	−0.0017 (15)	−0.0097 (15)
C13	0.0435 (15)	0.0476 (15)	0.0643 (18)	−0.0020 (12)	−0.0090 (13)	−0.0059 (14)
Cl1	0.0603 (4)	0.0411 (4)	0.0678 (5)	−0.0059 (3)	−0.0164 (3)	0.0108 (3)
Cl2	0.0692 (5)	0.0397 (4)	0.0607 (4)	−0.0071 (3)	−0.0095 (3)	−0.0059 (3)
Cl3	0.0596 (4)	0.0696 (5)	0.0525 (4)	−0.0197 (3)	−0.0093 (3)	0.0093 (4)
Cl4	0.0462 (4)	0.0542 (4)	0.0892 (5)	−0.0103 (3)	−0.0145 (4)	0.0059 (4)
N1	0.0438 (14)	0.0767 (16)	0.0514 (14)	0.0044 (12)	−0.0110 (11)	0.0038 (13)
N2	0.0462 (14)	0.0540 (14)	0.0671 (16)	0.0139 (11)	0.0006 (11)	0.0067 (12)
Ni1	0.03720 (18)	0.03082 (16)	0.04678 (19)	−0.00285 (13)	−0.00648 (13)	0.00183 (15)

### Geometric parameters (Å, °)

**Table:**

C1—N1	1.332 (3)	C8—H8A	0.9700
C1—C2	1.364 (3)	C8—H8B	0.9700
C1—H1	0.9300	C9—C13	1.381 (3)
C2—C3	1.381 (3)	C9—C10	1.385 (3)
C2—H2	0.9300	C10—C11	1.361 (3)
C3—C4	1.380 (3)	C10—H10	0.9300
C3—C6	1.496 (3)	C11—N2	1.330 (3)
C4—C5	1.358 (3)	C11—H11	0.9300
C4—H4	0.9300	C12—N2	1.330 (3)
C5—N1	1.326 (3)	C12—C13	1.370 (3)
C5—H5	0.9300	C12—H12	0.9300
C6—C7	1.505 (3)	C13—H13	0.9300
C6—H6A	0.9700	Cl1—Ni1	2.2487 (6)
C6—H6B	0.9700	Cl2—Ni1	2.2503 (6)
C7—C8	1.521 (3)	Cl3—Ni1	2.2760 (7)
C7—H7A	0.9700	Cl4—Ni1	2.2198 (7)
C7—H7B	0.9700	N1—H1A	0.8600
C8—C9	1.502 (3)	N2—H2A	0.8600
			
N1—C1—C2	120.2 (2)	C9—C8—H8B	108.1
N1—C1—H1	119.9	C7—C8—H8B	108.1
C2—C1—H1	119.9	H8A—C8—H8B	107.3
C1—C2—C3	119.8 (2)	C13—C9—C10	117.3 (2)
C1—C2—H2	120.1	C13—C9—C8	123.6 (2)
C3—C2—H2	120.1	C10—C9—C8	119.1 (2)
C4—C3—C2	117.6 (2)	C11—C10—C9	120.9 (2)
C4—C3—C6	118.3 (2)	C11—C10—H10	119.6
C2—C3—C6	124.0 (2)	C9—C10—H10	119.6
C5—C4—C3	120.9 (2)	N2—C11—C10	119.6 (2)
C5—C4—H4	119.6	N2—C11—H11	120.2
C3—C4—H4	119.6	C10—C11—H11	120.2
N1—C5—C4	119.6 (2)	N2—C12—C13	119.8 (2)
N1—C5—H5	120.2	N2—C12—H12	120.1
C4—C5—H5	120.2	C13—C12—H12	120.1
C3—C6—C7	117.56 (19)	C12—C13—C9	120.3 (2)
C3—C6—H6A	107.9	C12—C13—H13	119.9
C7—C6—H6A	107.9	C9—C13—H13	119.9
C3—C6—H6B	107.9	C5—N1—C1	121.9 (2)
C7—C6—H6B	107.9	C5—N1—H1A	119.1
H6A—C6—H6B	107.2	C1—N1—H1A	119.1
C6—C7—C8	110.12 (18)	C12—N2—C11	122.0 (2)
C6—C7—H7A	109.6	C12—N2—H2A	119.0
C8—C7—H7A	109.6	C11—N2—H2A	119.0
C6—C7—H7B	109.6	Cl4—Ni1—Cl1	98.27 (2)
C8—C7—H7B	109.6	Cl4—Ni1—Cl2	142.34 (3)
H7A—C7—H7B	108.2	Cl1—Ni1—Cl2	96.59 (3)
C9—C8—C7	116.96 (19)	Cl4—Ni1—Cl3	96.98 (3)
C9—C8—H8A	108.1	Cl1—Ni1—Cl3	134.16 (3)
C7—C8—H8A	108.1	Cl2—Ni1—Cl3	97.04 (3)
			
N1—C1—C2—C3	−0.1 (4)	C7—C8—C9—C10	−178.6 (2)
C1—C2—C3—C4	1.8 (4)	C13—C9—C10—C11	−2.5 (4)
C1—C2—C3—C6	−176.1 (2)	C8—C9—C10—C11	178.0 (2)
C2—C3—C4—C5	−2.0 (4)	C9—C10—C11—N2	0.8 (4)
C6—C3—C4—C5	176.0 (2)	N2—C12—C13—C9	−0.7 (4)
C3—C4—C5—N1	0.5 (4)	C10—C9—C13—C12	2.4 (4)
C4—C3—C6—C7	176.3 (2)	C8—C9—C13—C12	−178.1 (2)
C2—C3—C6—C7	−5.9 (4)	C4—C5—N1—C1	1.3 (4)
C3—C6—C7—C8	179.9 (2)	C2—C1—N1—C5	−1.5 (4)
C6—C7—C8—C9	178.7 (2)	C13—C12—N2—C11	−1.1 (4)
C7—C8—C9—C13	1.8 (3)	C10—C11—N2—C12	1.0 (4)

### Hydrogen-bond geometry (Å, °)

**Table:**

*D*—H···*A*	*D*—H	H···*A*	*D*···*A*	*D*—H···*A*
N1—H1A···Cl1^i^	0.86	2.43	3.150 (2)	142
N2—H2A···Cl3^ii^	0.86	2.25	3.114 (2)	178

Symmetry codes: (i) *x*+3/2, −*y*+1/2, *z*+1/2; (ii) −*x*−1/2, *y*+1/2, −*z*+1/2.
